# Identification of serum insulin-like growth factor binding protein 1 as diagnostic biomarker for early-stage alcohol-induced liver disease

**DOI:** 10.1186/1479-5876-11-266

**Published:** 2013-10-23

**Authors:** Heng-Hong Li, Kathryn Doiron, Andrew D Patterson, Frank J Gonzalez, Albert J Fornace Jr

**Affiliations:** 1 Department of Biochemistry and Molecular & Cellular Biology, Georgetown University, 3970 Reservoir Road, NW, New Research Building, Room E504, Washington, DC 20057, USA; 2 Department of Veterinary and Biomedical Sciences and The Center for Molecular Toxicology and Carcinogenesis, The Pennsylvania State University, University Park, PA 16802, USA; 3 Laboratory of Metabolism, Center for Cancer Research, National Cancer Institute, Bethesda, MD 20852, USA

**Keywords:** Alcohol-induced liver disease, Transcriptomics, Biomarker, *Igfbp1*, IGFBP1 protein

## Abstract

**Background:**

Alcohol consumption is a major cause of liver disease in humans. The use and monitoring of biomarkers associated with early, pre-clinical stages of alcohol-induced liver disease (pre-ALD) could facilitate diagnosis and treatment, leading to improved outcomes.

**Methods:**

We investigated the pathological, transcriptomic and protein changes in early stages of pre-ALD in mice fed the Lieber-Decarli liquid diet with or without alcohol for four months to identify biomarkers for the early stage of alcohol induced liver injury. Mice were sampled after 1, 2 and 4 months treatment.

**Results:**

Pathological examination revealed a modest increase in fatty liver changes in alcohol-treated mice. Transcriptomics revealed gene alterations at all time points. Most notably, the *Igfbp1* (Insulin-Like Growth Factor Binding Protein 1) was selected as the best candidate gene for early detection of liver damage since it showed early and continuously enhanced induction during the treatment course. Consistent with the microarray data, both *Igfbp1*mRNA expression in the liver tissue and the IGFBP1 serum protein levels showed progressive and significant increases over the course of pre-ALD development.

**Conclusions:**

The results suggest that in conjunction with other tests, serum IGFBPI protein could provide an easily measured biomarker for early detection of alcohol-induced liver injury in humans.

## Background

Alcohol consumption is responsible for about four percent of all disease world wide and is a major lifestyle-related cause of death in the United States [1], due in large part to complications from alcohol-induced liver disease (ALD). Both intrahepatic and extrahepatic stimuli triggered by alcohol abuse can cause ALD. Among the effects of interplay of these factors are metabolic dysfunctions such as fatty liver, obesity and excessive levels of hepatic free fatty acids, often followed by progressive pathologic changes such as inflammation, hepatomegaly and ultimately fibrosis and cirrhosis.

Diagnosis and stage-assessment of ALD provide guidance for treatment options. Diagnosis is presently based mainly on physical examination to determine general health and assessment of alcohol consumption history coupled with serum biomarker tests such as carbohydrate-deficient transferrin (CDT) [2] and various laboratory tests of liver function like aspartate aminotransferase/alanine aminotransferase ratio (AST/ALT) [3]. While it remains questionable whether serum CDT measurement reaches the specificity and sensitivity requirements for clinical use in the detection of chronic alcohol abuse [4], the liver function measurements can reflect liver damage directly. However there is at present no easily detectable biomarker to assess early stage changes prior to the appearance of later stage changes in liver function. Expensive and invasive techniques such as computerized tomography (CT) and magnetic resonance imaging (MRI) are frequently used to confirm the presence of steatosis and steatohepatitis. Liver biopsy is sometimes required to confirm correct diagnosis. Thus, there is a need for simple non-invasive and definitive markers of early changes in the development of ALD. Several have been proposed including serum beta-hexosaminidase [5,6], increased activity of superoxide dismutase (SOD) and neuron-specific enolase (NSE) in blood cells [7], increased levels of lymphocyte cytochrome P450 CYP2E1 [8]. A metabolomics study in mice revealed two metabolites in urine, *N*-acetylglutamine and *N*-acetylglycine, that might be useful markers for ALD [9]. Indole-3-lactic acid and phenyllactic acid may also be biomarker candidates for early stages of ALD [10].

Various animal models have been developed to characterize pathological changes of ALD. In particular, two protocols have been developed in mice that mimic aspects of ALD development in humans: the Lieber-DeCarli alcohol liquid diet [11,12] and the Tsukamoto-French intragastric alcohol feeding protocol [13,14]. Animals on the Lieber-DeCarli liquid diet display significant liver lesions including steatosis, apoptosis, structural alteration of mitochondria and endoplasmic reticulum and corresponding changes like those found early in human ALD. The Tsukamoto-French intragastric protocol produces severe liver damage including cirrhosis after a few months treatment. Certain genetically engineered mouse strains have also been used to investigate the development of ALD [15-17].

Here we investigated transcriptomic and pathological changes observed during early stage pre-ALD in mice fed the Lieber-Decarli liquid diet to identify biomarkers diagnostic for early stage pre-ALD. Transcriptomic approaches were previously used to profile changes in liver gene expression in mice and rats [18-21] and primates exposed to toxicants including ethanol [22,23]. The present study revealed the following: Chronic alcohol treatment for periods from one to four months led to a modest increase in fatty liver seen with standard techniques of histopathology. Transcriptomic analysis of these same livers revealed a gradual increase over time in the expression of several genes of which *Igfbp1* was chosen as the best candidate for further analysis because: 1) it showed high and consistent levels of alcohol-related increase; 2) the expression induction was observed even at the early time point; 3) IGFBP1 protein is liver-related and standard commercial immunological tests are available to detect its serum level in human samples. We suggest, therefore, that this marker, especially the protein, could be used as a diagnostic biomarker for early stage pre-ALD in humans. IGFBP1 is a member of the insulin-like growth factor binding protein (IGFBP) family and encodes a secreted protein with an IGFBP domain and a thyroglobulin type-I domain. The protein is strongly expressed in liver and kidney and circulates in the plasma.

## Methods

### Animals and treatments

Male mice (129/Sv strain, 6–8 weeks old) were fed *ad libitum* a liquid diet containing 4% ethanol (Lieber-DeCarli Diet, Dyets, Inc., Bethlehem, PA) as described [24]. Control animals were fed an isocaloric diet supplemented with maltose dextran (Dyets, Inc.). All mice were preconditioned on the control liquid diet for 7 days prior to switching half to the alcohol-containing arm. After one, two, or four months on the alcohol or control diets, six mice from each arm of the study, i.e. control and alcohol-fed, were killed in a CO_2_ chamber. These studies were approved by the Georgetown University Animal Care and Use Committee.

### Tissue collection and histology

Mice were sacrificed at the specified time points. Blood was collected by cardiac puncture immediately after the animals became unconsciousness. Blood samples were stored at room temperature for about 30 minutes to clot then centrifuged at 2000× g at 4°C for 10 minutes. Sera from each tube were collected and stored at −70°. Liver tissues were harvested and prepared for histology [24] by formalin-fixation and embedding in paraffin, sectioned and stained with hematoxylin and eosin. Histopathology was performed by a board-certified pathologist at Georgetown University Medical Center. Portions of liver tissue were snap-frozen for subsequent RNA analysis.

### RNA extraction

Up to 2 g of frozen liver tissue was homogenized in 5 ml Trizol reagent (Invitrogen, Carlsbad, CA), 1 ml of chloroform was added and the samples were centrifuged at 12,000 g for 15 min. The supernatant was collected and the samples were purified with the RNeasy Midi kit (Qiagen, CA). RNA quality was determined with an Agilent 2100 Bioanalyzer (Agilent Technologies, Santa Clara, CA) and the yield was measured with the Nanodrop 2000 (Thermo Scientific, Waltham, MA).

### Microarray analysis

Microarray analysis was carried out by standard procedures as described in the Agilent two-color expression array manual. Equal amounts of total RNA from each sample of one treatment group were combined to give about 100 mg total of which 16 μg was used for probe-labeling with the SuperScript indirect cDNA labeling system (Invitrogen) [25]. Probes were hybridized to mouse whole-genome 4x44K oligo microarrays (Agilent Technologies) following the manufacturer’s instructions. These arrays contain more than 41,000 unique mouse genes and transcripts. Two-color array RNA analysis was performed for each time point. The labeled alcohol-exposure and the respective control RNA were hybridized on one array and the pair was subjected to a reverse (dye-switching) fluorescence replicate. The software, Feature Extraction version 9.1 (Agilent Technologies) was used to filter, normalize, and calculate the signal intensities and Cy5/Cy3 ratios. The data discussed in this study were deposited in NCBI's Gene Expression Omnibus and are accessible through GEO Series accession number GSE44237 (http://www.ncbi.nlm.nih.gov/geo/query/acc.cgi?acc=GSE44237). These data were entered into a Rosetta Resolver v3.2 Gene Expression Data Analysis system (Rosetta Biosoftware, WA) for differential, hierarchical clustering, and trend-change analysis. The log(Ratio) was obtained by averaging the log(Ratio) of the reverse fluorescence pair from which the log(Ratio) error was calculated. Experiments for the different time courses were clustered using Genesis (http://genome.tugraz.at) with agglomerative algorithms to visualize time course dependent changes.

### Ingenuity pathway analysis (IPA)

Defined differential genes with changes in levels of more than 2 fold, either increased or decreased, were selected from each experiment. These gene-sets and corresponding expression values (ratios) for each time point were loaded into the IPA (Ingenuity Systems, Redwood City, CA) to generate a dataset. Biological functions and/or diseases that were most significantly associated with each set of data were identified using the Functional Analysis module. Fischer’s exact test was used to calculate a p-values to evaluate the probability that each biological function and/or disease assigned to that data set was not due to chance but resulted, in fact, from the treatment. Each gene in the dataset was mapped to its corresponding gene in the Ingenuity Pathways Knowledge Base to place it in a network of genes ('network generation'). These genes were then overlaid onto a global molecular network developed from information contained in the Ingenuity Pathways Knowledge Base. Networks for these focus genes were then algorithmically generated based on their connectivity.

### GEDI analysis

Gene Expression Dynamics Inspector (GEDI) [26] was used for analysis and visualization of patterns in the time-course microarray data. The genes that changed by more than two-fold at two or more time points were selected for GEDI analysis. The tiles in a two-dimensional mosaic generated by GEDI represent individual clusters in a self-organizing map (SOM), where the color of each tile is determined by the centroid value of a particular cluster [26]. The complete GEDI program package and details of the algorithm can be found at http://apps.childrenshospital.org/clinical/research/ingber/GEDI/gedihome.htm.

### Validation of levels of *Igfbp1* mRNA

Quantitative RT-PCR (qRT-PCR) assays were performed with a iCycler instrument (Bio-Rad, Hercules, CA), using iScript one-step RT-PCR kit probes (Bio-Rad). The primers and probe mix for the Taqman gene expression assay for *Igfbp1* were from ABI (Foster city, CA). β-actin was used as the internal control. qRT-PCR reactions were carried out in a total volume of 20 μl iScript kit instructions were followed for the thermocycling steps. A standard curve using serially diluted samples was made for each experiment. Data were analyzed with the iCycler software. The RNA levels of the target genes in the template were calculated from standard curves.

### ELISA

ELISA kits for measuring levels of IGFBP1 in serum were from Insight Genomics (Falls Church, VA). The measurement was performed according to the manufacturer’s instructions (https://www.insightgenomics.com/shop/datasheet/EK0383-protocol.pdf).

### Statistical analysis

Experimental values are presented as mean ± SD. Statistical analysis was performed using GraphPad Prism (San Diego, CA). The significance was determined using two-tailed student t-test. p-values of less than 0.05 were considered significant.

## Results

### General condition of mice

The daily consumption of the liquid diet and individual body weights were recorded for each mouse. No significant differences were observed between the control and treated mice, which had also been found by others using similar experimental protocols [24]. Liver tissues were subjected to hematoxylin and eosin (H&E) histopathology staining and Oil red O staining. The only alcohol-induced pathological difference observed in the livers at the two and four month time points was a slight increase in micro-vesicular fat deposition in the treated mice (Additional file 1: Figure S1, panels 1C to 1F and Additional file 2: Figure S2).

### Expression profiling

Transcriptomic changes in liver tissue associated with chronic alcohol consumption were evaluated in samples harvested after one, two, and four months of alcohol treatment. RNA samples from liver tissues pooled from six mice of each exposure group were analyzed. However, it should be noted that pooling may mask certain small changes in expression (see Discussion). For the microarray design, two-color arrays were used in which the labeled RNAs of controls and alcohol-exposures at each time point were hybridized on a single array. This approach reduces errors that can arise from inter-array variation. The reverse-fluorescence technical replication eliminates dye-bias that can occur in labeling.

**Figure 1:**
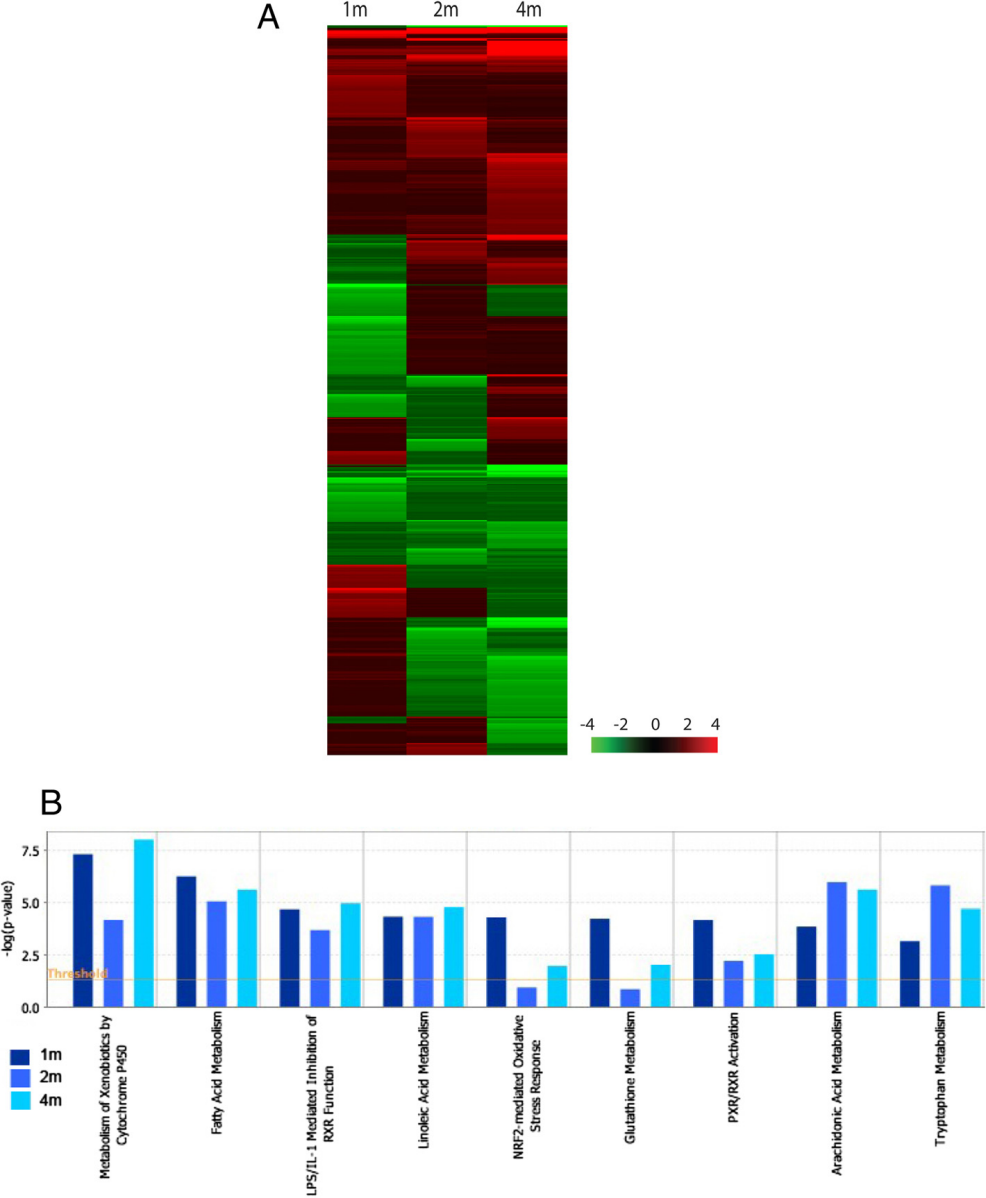
**Two dimensional clustering of differential genes in livers of alcohol-treated mice compared to liquid-diet control. (A)** Two dimensional clustering of differential genes in livers of alcohol-treated mice compared to liquid-diet control. Treatments for three time points are displayed, one-, two-, four-month. This clustering was derived from the ratio (treated/control) data. Red and green represented the levels of induction and repression of a particular gene respectively. The patterns of change were similar at two- and four-months. **(B)** Ingenuity Pathway Analysis results for differential genes. The orange line indicates the threshold of p-value of 0.05, i.e. -log_10_(p-value) of 1.3. Several metabolic pathways were modulated by alcohol treatment.

The quality of the RNA obtained from each liver was assessed and only RNA samples with an RIN (RNA integrity number) of at least 7.0 were pooled for microarray analysis. Dual color microarray analysis was performed to reveal changes in gene expression between the alcohol- and control-diet groups at each time point. In order to compare time-course effects of chronic alcohol consumption on the liver transcriptome, the microarray datasets from three time points were analyzed by two-dimensional clustering (Figure 1A). Expression of about 100 to 200 genes was increased by at least 2 fold and a roughly equal number showed a decreased expression at the three time points of exposure (Additional file 3: Table S1). On one hand, common genes were found among the three time points and, on the other hand, unique signatures occurred among the different time points. Eight hundred fifty-one sequences out of 41,000 unique mouse genes and transcripts had a change of more than 2 fold at one or more time points. These genes were selected for further Ingenuity pathway analysis.

**Figure 2:**
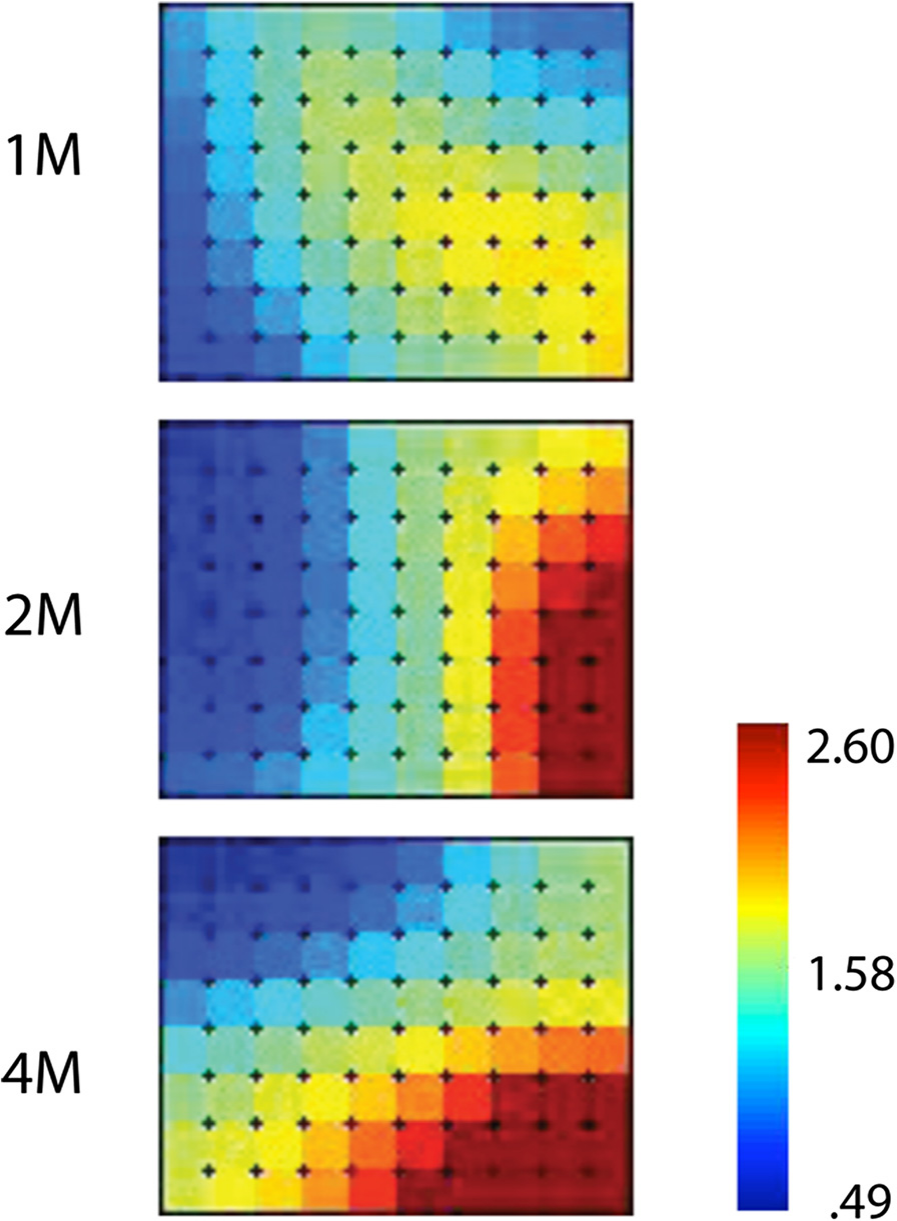
**Changes in gene expression during the four month treatment period as visualized with GEDI analysis using self-organizing maps (SOMs).** SOMs were constructed from data for 609 genes. Genes with the most robust induction were deep red while those most repressed were dark blue. Intermediate levels of change lie between these saturated colors.

The genes with altered expression were involved in several essential pathways including metabolism of fatty acids, linoleic acid and arachidonic acid and metabolism of xenobiotics by cytochromes P450 (Figure 1B). To visualize the changes in both induction and repression of gene expression, the genes that had a change of more than 2 fold at two or more time points were subjected to a GEDI analysis. The SOM matrix (Figure 2) shows the time-dependent effect of chronic alcohol intake on liver gene expression.

### Changes in gene expression

Two-dimensional clustering analysis revealed that the expression of certain genes was altered, either enhanced or repressed, at all time points in the alcohol treated animals. The “Trend” analysis in Rosetta Resolver was used to identify those signatures that showed progressively increased induction (These genes are listed in Additional file 4: Table S2).

Particular attention was paid to genes that are primarily expressed in liver. In particular, genes encoding proteins that can be readily identified in biofluids like blood, which would provide diagnostic simplification and were thus examined in more detail. *Igfbp1* is a gene that meets these criteria; it is expressed in liver, showed early and high levels of induction and its encoded protein, IGFBP1, can be readily quantified in blood by commercially available immunoassays. The microarray data showed that the expression level of *Igfbp1* in liver tissue from mice on the alcohol diet was increased by 1.9-, 3.9- and 6.8-fold, respectively, after one-, two-, or four- month’s alcohol treatment.

### Validation of microarray findings on *Igfbp1*

**Figure 3:**
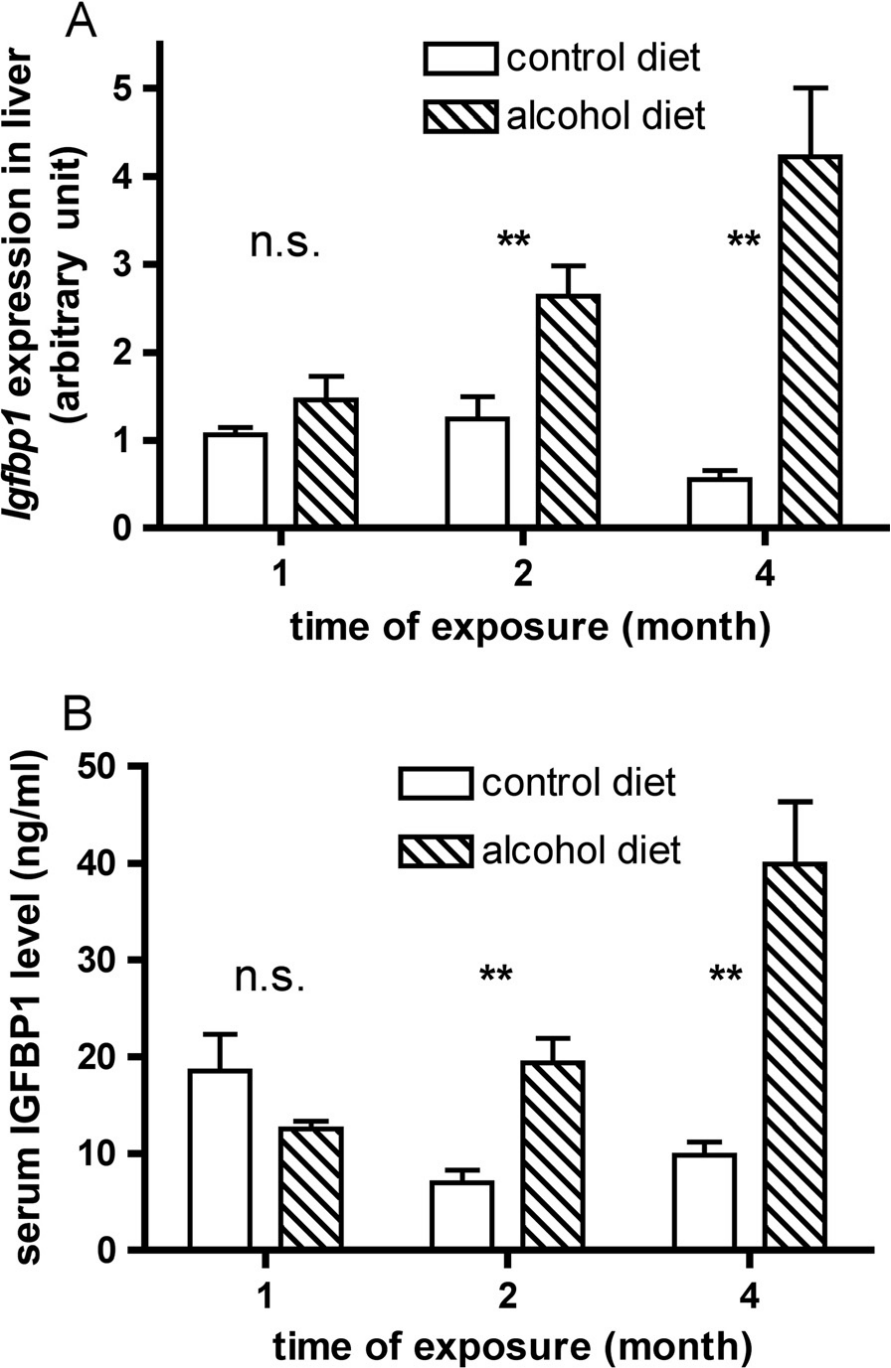
**Validation of results for mRNA and serum protein levels of IGFBP1. (A)** qRT-PCR was used to validate hepatic *Igfbp1* gene expression. The open bars were the control mice; black bars were for the alcohol-treated animals. **(B)** Serum levels of IGFBP1 protein measured by ELISA. Increases in the protein levels generally mimicked changes seen for microarray and qRT-PCT results. Data were for samples from individual mice, six animals in each group. For statistical significance, “n.s.” stands for no significance; “**” indicates a significant difference with a p-value less than 0.01.

To provide validation of the microarray data, qRT-PCR was performed on individual liver RNA samples. At two months and four months of alcohol consumption *Igfbp1* gene expression was significantly increased in liver tissue (Figure 3 upper panel). This result is consistent with the microarray data shown in Additional file 4: Table S2. Since IGFBP1 protein is known to be in blood, protein levels were measured in mouse sera by ELISA (Figure 3 lower panel). Alcohol treatment for two and four months significantly increased the levels (p-value of 0.002 and 0.001 respectively). The dot plot of serum IGFBP1 measurement is shown in Additional file 5: Figure S3 to display the individual variation of the tested samples. The changes in gene expression and protein level at the one-month time point were not statistically significant. At the later two time points, the increases in gene expression were similar to those seen for protein levels. For reasons not known, IGFBP1 protein levels in control groups at different time points varied to certain extent. At two-month serum IGFBP1 decreased by around 50% when compared with that of one-month control while the liver IGFBP1 mRNA expression level did not change significantly. At four-month both liver expression and serum protein level of IGFBP1 of control group are significantly lower than that of one-month control. Nevertheless alcohol feeding remarkably increased IGFBP1 on both liver mRNA expression and serum protein level, which are shown in measurements in Figure 3 and supported by the t-test results in Additional file 6: Table S3.

## Discussion

Transcriptomic analysis was applied to screen for markers of early-stage pre-ALD in mice treated for up to four months with 4% alcohol by liquid diet. *Igfbp1* was identified as a particularly useful biomarker gene for alcohol-induced disease because its expression is liver-related, and expression increased significantly and progressively over the course of treatment. Furthermore IGFBP1 protein, which is readily detectable in mouse or human serum by a commercially available ELISA assays, also increased significantly and thus may be to be a good candidate for clinical use in humans.

Transcriptomics has been used to analyze changes in gene expression in liver associated with alcohol treatment of several species including mice and rats [18-23]. Although the pathways affected were generally the same from study to study, differences in feeding systems and other experimental parameters make it difficult to directly compare results. For instance the changes in gene expression were very different in two rat strains treated in different ways, i.e., Sprague–Dawley rats employing voluntary alcohol intake and Wistar rats treated via intragastric introduction of alcohol [27]. In the present study, induction of genes involved in the metabolism of fatty acids, lipids, nucleotides, and glutathione, as well as metabolism of xenobiotics, were observed, consistent with previous publications. These results are similar to the gene-profiling study in the C57Bl/6 mouse model of four weeks’ intragastric ethanol infusion [18]. We observed changes of pathways and also genes such as induction of glutathione S-transferases and cytochromes P450. In the cytochromes P450 family, the specific members including *Cyp2a4*, *Cyp2c29*, and *Cyp2b9* showed induction by alcohol in both studies.

In this study transcriptomics profiling was performed on pooled samples from each group to obtain the differentially expressed genes. Statistical implications of such sample pooling have been discussed [28-30]. In theory, if individual variations in levels of gene expression have a normal distribution then pooling samples could serve to reduce the variance [28,29]. On the other hand, pooling may hide values for outliers so as to cause loss of actual information. In a study of rat liver responses to various toxic agents, the transcriptomics microarray results were compared for the pooled data and for individual samples and about 90 percent of the transcript changes seen in the pooled samples were also found in the individual samples [30]. However, 50% more expression changes were observed in the individual samples than were observed in the pooled samples, although most of these excess changes were subtle. The objective of this study was to identify biomarkers that are robustly altered by alcohol intake. In addition, subtle gene expression changes are usually associated with low reproducibility. Therefore, we applied the sample pooling method with the understanding that we might miss some genes that were modestly altered by alcohol, but which allowed us to narrow down the search for the genes with more robust changes. To assess the individual variation of the selected biomarker, individual liver RNA and serum samples were used for gene expression and serum protein validation assays (Figure 3).

We here showed the association of early pre-ALD conditions with increased *Igfbp1* expression and increased levels of IGFBP1 protein in serum. This protein is strongly expressed in liver and kidney and circulates in the plasma. The association of alcohol intake and increased levels of IGFBP1 was reported in rodents and humans, primarily after acute exposure [31-34]. Several studies showed an acute increase in levels of IGFBP1 in normal human subjects and in alcohol abusers within 8 hours of alcohol ingestion [32-34]. In a study in humans, IGFBP1 levels were increased in human serum after a single dose of alcohol had been administration to nine healthy volunteers [31]. In patients with alcoholic liver cirrhosis and ascites, the IGF affinity profile of the IGFBP family was found markedly altered [35]. However from these human studies, it remained unclear whether increased levels of IGFBP1 persisted with chronic alcohol consumption and if it did, whether levels continue to increase in parallel with the development of ALD.

Only two studies were found on regulation and levels of IGFBP1 with chronic alcohol exposure in animals [36,37]. In rodents chronically exposed to alcohol, the rats were fed on alcohol-agar blocks that contained over 30% alcohol. The doses used far exceeded those possible in human consumption. In addition, only one time-point was used in these studies, which made it impossible to assess the development and progress of chronic alcohol induced liver pathological changes and to address how *Igfbp1* expression change associated with the progress. In our study, a four-month liquid diet feeding was carried out with 4% alcohol and samples collected after one, two and four months. The concentration of our feeding scheme was closer to the consumption of chronic alcoholics in human. The mRNA expression and the serum protein levels of IGFBP1 increased gradually along the time course of the alcohol exposure. The finding of lower serum IGFBP1 levels associated with nonalcoholic fatty liver disease [38] suggests that increased IGFBP1 expression may be caused by alcohol consumption.

## Conclusion

Hepatic expression of *Igfbp1* gene and serum level of IGFBP1 protein were found progressively increased during the four months’ treatment course. Histopathology showed mild fat deposition in alcohol-fed mice. The results indicate that in conjunction with other tests, IGFBP1 protein in serum could provide an easily measured biomarker for early detection of pre-ALD.

## Abbreviations

ALD: Alcohol-induced liver disease; pre-ALD: Pre-clinical stages of alcohol-induced liver disease; IGFBP1/Igfbp1: Insulin-like growth factor binding protein 1; CT: Computerized tomography; MRI: Magnetic resonance imaging; SOD: Superoxide dismutase; NSE: Neuron-specific enolase; GEDI: Gene expression dynamics inspector; SOM: Self-organizing map; qRT-PCR: Quantitative reverse transcription PCR; ELISA: Enzyme-linked immunosorbent assay.

## Competing interests

The authors declare that they have no competing interests.

## Authors' contributions

HHL was responsible for experimental design, data collection, data analysis, literature search and drafting the manuscript. KD was responsible for animal care protocol development and treatment. AJF was the principle investigator and was responsible for obtaining funding and study supervision. AJF, ADP, and FJG provided input on study design, data interpretation, and manuscript preparation. All authors critically reviewed the manuscript before its final version. All authors read and approved the final manuscript.

## Supplementary Material

Additional file 1: Figure S1 Liver histology (HE staining) of wild-type 129Sv mice after control (A, C, & E) and 4% alcohol containing (B, D, & F) liquid diet. Click here for file

Additional file 2: Figure S2 Oil red O staining of liver samples. Click here for file

Additional file 3: Table S1 Number of signatures with the fold change of more than 2 with p-value less than 0.05. Click here for file

Additional file 4: Table S2 The fold change of the genes that show consistently increased induction along the alcohol treatment course. Click here for file

Additional file 5: Figure S3 Dot plot of the serum IGFBP1 protein levels. Click here for file

Additional file 6: Table S3 T-test of IGFBP1 level validation results. Click here for file
