# Supplementary material for: Identification of serum insulin-like growth factor binding protein 1 as diagnostic biomarker for early-stage alcohol-induced liver disease
Source: J Transl Med. 2013 Oct 23;11:266. doi: 10.1186/1479-5876-11-266 (PMC4016206; doi:10.1186/1479-5876-11-266)
Supplement: Additional file 1: Figure S1 — Liver histology (HE staining) of wild-type 129Sv mice after control (A, C, & E) and 4% alcohol containing (B, D, & F) liquid diet. [file 1479-5876-11-266-S1.pdf]

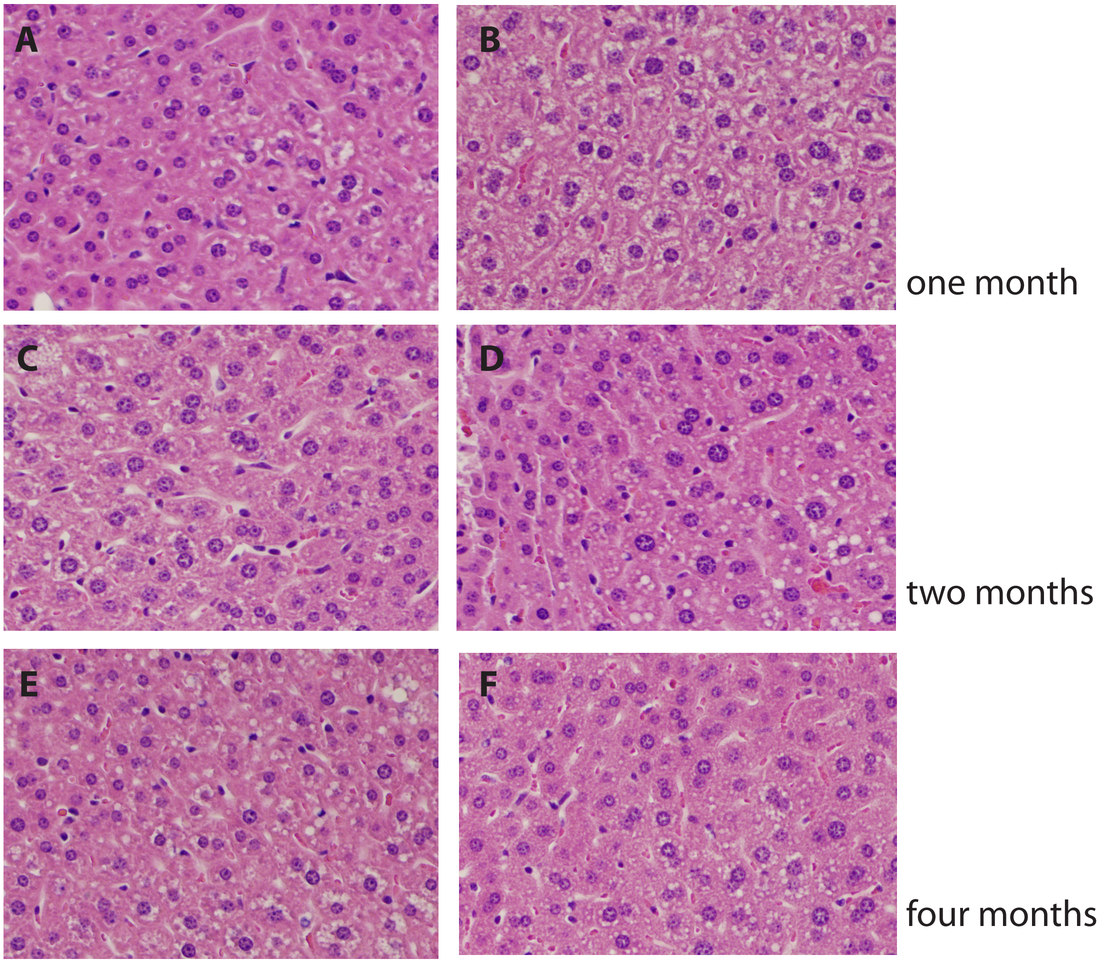

Supplement figure 1 Liver histology (HE staining) of wild-type 129Sv mice after control (A, C, & E) and 4% alcohol containing (B, D, & F) liquid diet.
