# Supplementary figures and images for: Identification of serum insulin-like growth factor binding protein 1 as diagnostic biomarker for early-stage alcohol-induced liver disease
Source: J Transl Med. 2013 Oct 23;11:266. doi: 10.1186/1479-5876-11-266 (PMC4016206; doi:10.1186/1479-5876-11-266)

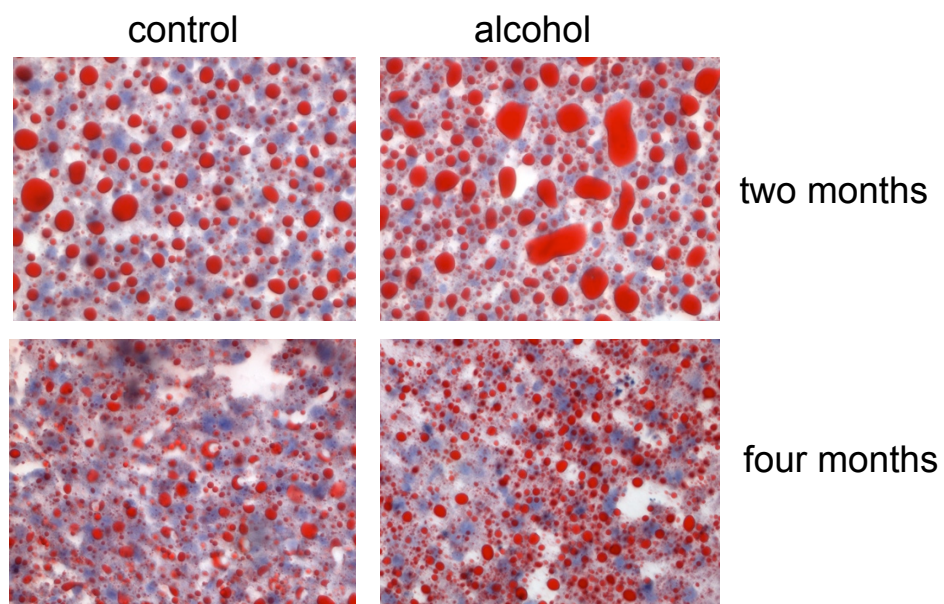

Supplement figure 2. Oil red O staining of liver samples

Supplement: Additional file 2: Figure S2 — Oil red O staining of liver samples. [file 1479-5876-11-266-S2.pdf]

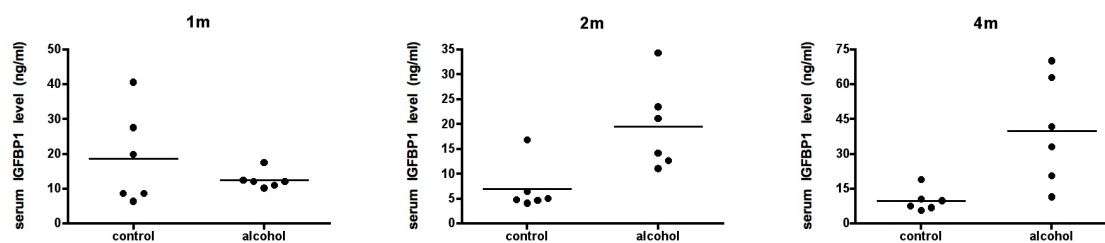

Supplement figure 3. Dot plot of the serum IGFBP1 protein levels.

Supplement: Additional file 5: Figure S3 — Dot plot of the serum IGFBP1 protein levels. [file 1479-5876-11-266-S5.pdf]
