# Supplementary material for: Identification of serum insulin-like growth factor binding protein 1 as diagnostic biomarker for early-stage alcohol-induced liver disease
Source: J Transl Med. 2013 Oct 23;11:266. doi: 10.1186/1479-5876-11-266 (PMC4016206; doi:10.1186/1479-5876-11-266)
Supplement: Additional file 3: Table S1 — Number of signatures with the fold change of more than 2 with p-value less than 0.05. [file 1479-5876-11-266-S3.pdf]

Supplement Table 1 Number of signatures with the fold change of more than 2 with p-value less than 0.05

	Induction	Repression
One month	158	187
Two month	115	94
Four month	216	165
