# Supplementary material for: Identification of serum insulin-like growth factor binding protein 1 as diagnostic biomarker for early-stage alcohol-induced liver disease
Source: J Transl Med. 2013 Oct 23;11:266. doi: 10.1186/1479-5876-11-266 (PMC4016206; doi:10.1186/1479-5876-11-266)
Supplement: Additional file 4: Table S2 — The fold change of the genes that show consistently increased induction along the alcohol treatment course. [file 1479-5876-11-266-S4.pdf]

Supplement Table 2. The fold change of the genes that show consistently increased induction along the alcohol treatment course

Primary Sequence Name	Description	One Month	Two Months	Four Months
Cyp2b10	cytochrome P450, family 2, subfamily b, polypeptide 10	3.50	7.00	14.12
Gadd45b	growth arrest and DNA-damage-inducible 45 beta	1.34	5.40	9.57
Igfbp1	insulin-like growth factor binding protein 1	1.66	3.89	6.67
Cyp2c29	cytochrome P450, family 2, subfamily c, polypeptide 29	1.69	1.76	5.43
Pnliprp1	pancreatic lipase related protein 1	1	1.06	5.20
Cyp3a16	cytochrome P450, family 3, subfamily a, polypeptide 16	2.39	2.47	5.19
Reg3d	regenerating islet-derived 3 delta	1	1.32	5.01
Cyp3a11	cytochrome P450, family 3, subfamily a, polypeptide 11	2.21	2.44	4.94
Cyp3a44	cytochrome P450, family 3, subfamily a, polypeptide 44	2.00	2.10	4.82
Fmo3	flavin containing monooxygenase 3	1.09	1.05	4.65
1810049H19Rik	RIKEN cDNA 1810049H19 gene	1.04	1.01	4.60
Reg2	regenerating islet-derived 2	1	-1.05	4.48
Rgs1	regulator of G-protein signaling 1	2.05	1.78	4.43
Cyp2b9	cytochrome P450, family 2, subfamily b, polypeptide 9	1.40	3.89	4.34
Cpb1	carboxypeptidase B1 (tissue)	1	1.03	4.33
Cyp3a41a	cytochrome P450, family 3, subfamily a, polypeptide 41A	2.14	2.20	4.22
Lhfpl1	lipoma HMGIC fusion partner-like 1	1.03	-1.29	3.95
Cubn	cubilin (intrinsic factor-cobalamin receptor)	1	1.00	3.95
Cyp2c39	cytochrome P450, family 2, subfamily c, polypeptide 39	1.38	2.10	3.59
Cyp2a5	cytochrome P450, family 2, subfamily a, polypeptide 5	2.12	2.00	3.48
Abcd2	ATP-binding cassette, sub-family D (ALD), member 2	1.22	1.82	3.27
Jun	Jun oncogene	1.10	2.52	3.27
Olfr1228	olfactory receptor 1228	1.20	1.51	3.26
Zfp583	zinc finger protein 583	1	1.08	3.20
4930430M16Rik	RIKEN cDNA 4930430M16 gene	1	1.00	3.12
B930079E06	hypothetical protein B930079E06	1.04	1.00	3.11
LOC620760	hypothetical protein LOC620760	1	1.48	3.07
1500002O10Rik	RIKEN cDNA 1500002O10	1	1.05	2.95

	gene			
4930444P10Rik	RIKEN cDNA 4930444P10 gene	1.06	1.48	2.92
V1rc12	vomeronasal 1 receptor, C12	1	1.04	2.90
1700121L16Rik	RIKEN cDNA 1700121L16 gene	1	1.00	2.67
Dcpp2	demilune cell and parotid protein 2	1	1.16	2.64
Prss3	protease, serine, 3	1.12	-1.03	2.63
4930570B17Rik	RIKEN cDNA 4930570B17 gene	1	-1.08	2.62
2610010G17Rik	RIKEN cDNA 2610010G17 gene	1	1.32	2.56
9330154F10Rik	RIKEN cDNA 9330154F10 gene	1	1.00	2.54
Thbd	thrombomodulin	1.05	1.39	2.52
C030039E19Rik	RIKEN cDNA C030039E19 gene	1	1.00	2.50
Cyp2c38	cytochrome P450, family 2, subfamily c, polypeptide 38	1.57	2.21	2.48
Epdr1	ependymin related protein 1 (zebrafish)	1.12	-1.03	2.46
Cyp2a22	cytochrome P450, family 2, subfamily a, polypeptide 22	1.34	1.32	2.44
Olfr1324	olfactory receptor 1324	1	-1.28	2.43
Aspn	asporin	1	1.00	2.40
Serpinb9b	serine (or cysteine) peptidase inhibitor, clade B, member 9b	1	1.29	2.39
S100a8	S100 calcium binding protein A8 (calgranulin A)	1.30	1.02	2.38
Cyp4a10	cytochrome P450, family 4, subfamily a, polypeptide 10	2.01	2.51	2.38
Cyp2c37	cytochrome P450, family 2. subfamily c, polypeptide 37	1.22	1.55	2.38
Trpm8	transient receptor potential cation channel, subfamily M, member 8	1	-1.05	2.37
Trex2	three prime repair exonuclease 2	1.43	1.49	2.37
Smgc	submandibular gland protein C	1	1.19	2.36
AF529169	cDNA sequence AF529169	1	-1.39	2.35
1110014L15Rik	RIKEN cDNA 1110014L15 gene	1	1.85	2.35
Adrb2	adrenergic receptor, beta 2	1	1.45	2.30
D17H6S56E-3	DNA segment, Chr 17, human D6S56E 3	1	-1.05	2.26
9430099H24Rik	RIKEN cDNA 9430099H24 gene	1	1.00	2.22
Dusp8	dual specificity phosphatase 8	1	1.08	2.21
Gstt3	glutathione S-transferase, theta 3	1.34	1.27	2.21
B230104C08Rik	RIKEN cDNA B230104C08 gene	1	1.25	2.18

ENSMUSG00000054651	predicted gene, ENSMUSG00000054651	1	1.00	2.18
A430090E18Rik	RIKEN cDNA A430090E18 gene	1	1.00	2.16
Rnase1	ribonuclease, RNase A family, 1 (pancreatic)	1.31	1.04	2.16
A330049M09Rik	RIKEN cDNA A330049M09 gene	1	-1.20	2.16
ENSMUSG00000053412	predicted gene, ENSMUSG00000053412	1.02	-1.23	2.14
4930426L09Rik	RIKEN cDNA 4930426L09 gene	1	1.22	2.13
Olfr619	olfactory receptor 619	1	-1.12	2.13
1700022E09Rik	RIKEN cDNA 1700022E09 gene	1	1.39	2.12
4933425B07Rik	RIKEN cDNA 4933425B07 gene	1	1.25	2.10
Olfr670	olfactory receptor 670	1	1.00	2.09
D930043O14Rik	RIKEN cDNA D930043O14 gene	1	-1.10	2.09
A630080F05Rik	RIKEN cDNA A630080F05 gene	1	1.03	2.08
BC057022	cDNA sequence BC057022	1	1.07	2.08
EG432743	predicted gene, EG432743	1.04	1.65	2.06
BC037438	cDNA sequence BC037438	1	-1.37	2.06
AI839979	expressed sequence AI839979	1	1.53	2.05
Tph2	tryptophan hydroxylase 2	1	1.09	2.04
C130034I18Rik	RIKEN cDNA C130034I18 gene	1	-1.28	2.04
Ccl6	chemokine (C-C motif) ligand 6	1.23	1.51	2.03
Snap91	synaptosomal-associated protein 91	1	-1.15	2.03
6430500C12Rik	RIKEN cDNA 6430500C12 gene	1	1.00	2.03
ENSMUSG00000053880	predicted gene, ENSMUSG00000053880	1.17	1.00	2.02
Mcm6	minichromosome maintenance deficient 6 (MIS5 homolog, S. pombe) (S. cerevisiae)	1.31	1.20	2.02
ENSMUSG00000053802	predicted gene, ENSMUSG00000053802	1	1.72	2.01
Aplp1	amyloid beta (A4) precursor-like protein 1	1	2.08	1.85
