# Supplementary material for: Identification of serum insulin-like growth factor binding protein 1 as diagnostic biomarker for early-stage alcohol-induced liver disease
Source: J Transl Med. 2013 Oct 23;11:266. doi: 10.1186/1479-5876-11-266 (PMC4016206; doi:10.1186/1479-5876-11-266)
Supplement: Additional file 6: Table S3 — T-test of IGFBP1 level validation results. [file 1479-5876-11-266-S6.pdf]

Supplement Table 3. T-test of IGFBP1 level validation results

A. T-test results of liver IGFBP1 mRNA measurements by qRT-PCR

p-value	2m_control	4m_control	1m_ethanol	2m_ethanol	4m_ethanol
1m_control	0.90	0.002	0.95	n.a.	n.a.
2m_control		0.02	n.a.	0.005	n.a.
4m_control	0.02		n.a.	n.a.	0.0003
2m_ethanol	0.005	n.a.	0.005		0.08
4m_ethanol	n.a.	0.0003	0.002	0.08	

B. T-test results of serum IGFBP1 protein measurements by ELISA

p-value	2m_control	4m_control	1m_ethanol	2m_ethanol	4m_ethanol
1m_control	0.008	0.04	0.13	n.a.	n.a.
2m_control		0.14	n.a.	0.0002	n.a.
4m_control	0.14		n.a.	n.a.	0.0001
2m_ethanol	0.0002	n.a.	0.01		0.007
4m_ethanol	n.a.	0.0001	0.0003	0.007	

p-value less than 0.05 is considered statistically significant. The grids corresponding to the same group are filled in grey. “n.a.” means that t-test result is not available for this pair since the comparison is not biologically meaningful.
